# Human Weights

**Published:** 1875-02

**Authors:** 


### HUMAN WEIGHTS.

If every human being in the world was weighed, the average weight would be about 100 lbs. avoirdupois. The average weight of men at 40 is 140 lbs.; the average weight of women at 50 is 130 lbs. Average weight of babies at birth is 7 lbs., and yet at two pounds they have been known to live. Young men at 20 average 135; young women at 20 average 110.

Most persons want to weigh more than they do, although it is known that the heavier persons are the less chance they have for health, and the greater chance for a premature death. Thin men and thin women live on an average quite a number of years longer than the rubicund and robust, and thin women, weighing 100 lbs., and a man at thirty-five weighing 135 lbs., will average ten years longer life than if their respective weights were one-fourth greater.

A youth of twenty years died lately, weighing 450 lbs. Over-grown men, giants, never live long, often dying of consumption before 40.

Fat people are never well; they seem to be so, and they themselves feel well and think they are well, but close inquiry will show that there is some unnatural condition in the body itself or some of its functions, a running sore, some breaking out on the skin, some nasal discharge, some mattering of the eyelids; or if none of these, they die suddenly and prematurely. "Fleshy people," as we call them, or more properly "fatty" people, have an excess of fat, and this excess makes them more liable to disease and an untimely death.

